# Usability Testing of a Mobile App to Report Medication Errors Anonymously: Mixed-Methods Approach

**DOI:** 10.2196/12232

**Published:** 2018-12-21

**Authors:** Doris George, Mohamed Azmi Hassali, Amar-Singh HSS

**Affiliations:** 1 Social & Administrative Pharmacy School of Pharmaceutical Sciences Universiti Sains Malaysia Penang Malaysia; 2 Clinical Research Center Department of Paediatrics Raja Permaisuri Bainun Hospital Ipoh Malaysia

**Keywords:** mobile app, usability, medication error reporting, anonymous

## Abstract

**Background:**

Reporting of medication errors is one of the essential mechanisms to identify risky health care systems and practices that lead to medication errors. Unreported medication errors are a real issue; one of the identified causes is a burdensome medication error reporting system. An anonymous and user-friendly mobile app for reporting medication errors could be an alternative method of reporting medication error in busy health care settings.

**Objective:**

The objective of this paper is to report usability testing of the Medication Error Reporting App (MERA), a mobile app for reporting medication errors anonymously.

**Methods:**

Quantitative and qualitative methods were employed involving 45 different testers (pharmacists, doctors, and nurses) from a large tertiary hospital in Malaysia. Quantitative data was retrieved using task performance and rating of MERA and qualitative data were retrieved through focus group discussions. Three sessions, with 15 testers each session, were conducted from January to March 2018.

**Results:**

The majority of testers were pharmacists (23/45, 51%), female (35/45, 78%), and the mean age was 36 (SD 9) years. A total of 135 complete reports were successfully submitted by the testers (three reports per tester) and 79.2% (107/135) of the reports were correct. There was significant improvement in mean System Usability Scale scores in each session of the development process (*P*<.001) and mean time to report medication errors using the app was not significantly different between each session (*P*=.70) with an overall mean time of 6.7 (SD 2.4) minutes. Testers found the app easy to use, but doctors and nurses were unfamiliar with terms used especially medication process at which error occurred and type of error. Although, testers agreed the app can be used in the future for reporting, they were apprehensive about security, validation, and abuse of feedback featured in the app.

**Conclusions:**

MERA can be used to report medication errors easily by various health care personnel and it has the capacity to provide feedback on reporting. However, education on medication error reporting should be provided to doctors and nurses in Malaysia and the security of the app needs to be established to boost reporting by this method.

## Introduction

Patient safety incident is defined as a situation that resulted or did not result in unnecessary harm to a patient due to the health care process, procedures, or medications given to the patient. Harm to patient can be further classified based on type of harm and extent of harm, including social and economic implications [1]. The theme of the third Global Patient Safety Challenge launched in 2017 by the World Health Organization (WHO) is medication safety [2]. The WHO reports that all medication errors potentially can be avoided by improving health care systems and practices of medication ordering, prescribing, preparing, dispensing, administering, and monitoring. Therefore, all health care personnel involved in any medication process should be committed to continuous improvement in health care systems and practices.

Medication error reporting is one of the essential mechanisms to identify risky health care systems and practices, and information regarding medication errors should be shared among health care professionals for learning purposes and prevention of further errors [3]. In Malaysia, medication error reports to the national database revealed reports of medication errors were substantially by pharmacists (98%) with 76% of the medication error reports involving the prescribing process [4]. In the United States, 80% of hospitals estimated that only a few adverse event reports were reported by doctors [5]. This indicates there are unreported medication errors from certain professions such as doctors. Every unreported medication error is a chance lost to identify trends, risky systems, and practices for improving health care [6]. Encouraging various groups of health personnel in medication error reporting would give a better perception of medication error occurrences at the institution.

The roadblocks in medication error reporting can be divided into three major categories based on recent literature reviews: attitudes of reporters, the error involved, and the reporting system [7,8]. Attitudes of reporters include fear of impending actions as a result of reporting and simply not seeing the need of reporting. Error severity also influences reporting. A reporting system that is laborious along with lack of education on reporting, nonsupportive management, and lack of feedback discourages reporting. The reporting system is a modifiable category that can be improved to encourage reporting. This leads to the idea of creating a medication error reporting method that is user-friendly, a fast mode of reporting, requires little training to use, is available at all times, preserves anonymity, and—importantly—is able to provide feedback on a large scale instantaneously.

Therefore, the aim of this paper is to report the usability testing of a mobile app for reporting medication errors with the ultimate aim to design an anonymous, user-friendly app for reporting medication errors.

## Methods

### Study Design

Usability is a measure of how easy a product such as website or app is to use. It can also be defined as methods for improving ease of use during the design process as described by Jakob Nielsen [9]. This usability testing involved a mixed methodology of both quantitative and qualitative data collection conducted from January 2018 to March 2018. Task performance and rating of app methods were used to retrieve quantitative data. Qualitative data were retrieved through focus group discussion methods on completion of tasks. A series of three sessions were conducted in the meeting room of the hospital where a hotspot was created for internet connectivity.

### Medication Error Reporting App

The design of the Medication Error Reporting App (MERA) was developed by an independent pharmacist to run on two mobile phone operating platforms, the iOS and Android. Content of MERA was adapted from the current Medication Error Reporting System Form (BPF/104/ME/02) in Malaysia. Based on analysis of the current reporting database (2013-2015), common missing and incorrect information were recorded. Two public hospital verifiers of medication error reports were interviewed to discuss the content of MERA. Changes incorporated into MERA based on these are explained here.

Domains that were appended were location of error (inpatient or outpatient for errors that occur at hospital settings), initial medication process that error occurred (labeling, filling, preparing, and monitoring), types of error (subcategorized as shown in Multimedia Appendix 1), and possible contributing factors (categorized based on an extensive literature search as shown in Multimedia Appendix 2).

The age of patient domain was categorized into neonates, infants, children, adolescents, adults, and geriatrics because data in this category were mostly missing from the current reports.

A drop-down list of medication available for Ministry of Health (MOH) use for quick entry was also incorporated into the app.

Information on type and size of container and manufacturer details are not included in MERA. Features to upload images such as a prescription or a photo of the label and any other relevant materials were not included due to cost implications.

### Selection of Testers

#### Testers’ Characteristics

Testers were selected based on potential users of MERA in the MOH, which included doctors, pharmacists, and nurses. Testers were conveniently selected from a large 990-bed public hospital, Raja Permaisuri Bainun Hospital, in Ipoh, Malaysia. Testers included both experts in the field of medication error reporting or related works and users or novices of the current medication error reporting form or website. Testers were categorized into two categories: (1) user or novice and (2) expert or nonexpert. Experts for the study were defined as health care professionals who encounter medication errors in practice and are involved in patient safety meetings for department, facility, or state, or are involved in medication error-related research. Users were defined as health care professionals who have reported medication errors using manual forms more than once in the past year or are involved in verifying medication error reports for facility or state. Health care professionals who have their subordinates fill in the medication error reports were considered novice.

Other criteria for selecting testers included those that owned mobile phones with an iOS or Android operating system and had been using it for not less than 3 months.

#### Sample Size

It has been concluded that five testers are typically enough to discover 80% of the problems in a test [10] and 15 testers is enough to discover 90% of the problems in a test [11]. In this study, the sample size for each session was set at 15 to obtain 90% of the problems encountered with MERA. Assuming more than two sessions of testing would be required to obtain a usability score for the app, power analysis was conducted using G*Power version 3.1.9.2 software [12] by setting 80% power to detect the difference among means versus the alternative of equal means using an *F* test with a .05 significance level. A total calculated sample size of nine was obtained by assuming the standard deviation to be 5, expected usability score to rise from 60% to 80%, and the calculated effect size to be 1.63. Similarly, by assuming mean time to complete a medication error report reduced from 10 minutes to 5 minutes, a standard deviation of 5, and calculated effect size of 1.03, the minimum sample size required was 15.

### Testing Procedure

#### Procedure 1

The testers were briefed on the background and purpose of the app before starting the session. Consent was obtained from each tester prior to starting the sessions and basic demographic data such as profession, age, and gender were recorded. Testers for each session were all different.

#### Procedure 2

Each tester downloaded MERA by scanning a quick response (QR) code to retrieve the app onto their mobile phones and were given time to go through the app. MERA has two major functions: to report medication errors and to provide feedback of medication error reports. Testers were presented with three medication error scenarios involving medication errors initiated in three main medication processes (prescribing, administrating, and dispensing) for reporting (Multimedia Appendix 3). The scenarios were randomly selected from real cases reported from the hospital. They were required to read the scenarios and submit medication error reports using the app. During the task, the problems encountered and the step(s) testers sought help for were evaluated. Testers were told to record the time they attempted to fill in the report and the time they completed submission of the report using MERA. Immediately after the testers completed submitting the three reports, they were asked to rate the perceived usability of MERA based on the System Usability Scale (SUS; Multimedia Appendix 4).

#### Procedure 3

Once the testers completed rating using the SUS, a focus group discussion was conducted to discuss the challenges and problems encountered using the app, any good points regarding the app, suggestions to improve the app, and potential use of MERA by all health care professionals. A checklist of focus group discussion question points was used to conduct the session (Multimedia Appendix 5). The focus group discussions were conducted by the same researcher, who is a practicing hospital pharmacist and has experience in reporting medication errors, compiling medication error reports for the state, and is involved in various research involving medication errors. This researcher also underwent qualitative interview training.

Evaluation and redesign of app functions and interface were done based on the feedback obtained during the discussions.

### Tools and Data Collection

#### Quantitative Data

The quantitative data collected consisted of time taken to complete the medication error report, total medication error reports successfully submitted, number of incorrect reports submitted, and the SUS score to measure perceived usability.

The SUS is a validated tool that is simple and easy to evaluate how one perceives the usability of a system or app [13]. The SUS is a set of 10 questions with a Likert-scale rating of 1=strongly disagree to 5=strongly agree. The scale of odd questions (1, 3, 5, 7, and 9) are deducted by 1, whereas for even questions (2, 4, 6, 8, and 10), 5 is deducted from the scale. The SUS score calculation is done by summing the modified scale and multiplying it by 2.5; the score has a range of 0 to 100. For a score higher than 80.3, the app is considered excellent, a score of 80.2 to 74 considers the app is usable, a score of 73.9 to 68 considers the app is usable but could improve, a score of 67.9 to 51.9 considers improvement is recommended, and a score of 51 or less considers the app should be fixed. For this study, improvement and usability testing was done until a score of 74 or higher was achieved.

#### Qualitative Data

Themes for questions used in the focus group discussion were derived from seven theoretical domains frameworks as suggested in a literature search [14,15]: usability, visual design and layout, content, potential user engagement, security, validation of report, and other comments. A semistructured question guide was prepared as a checklist to ensure all topics were covered and probing questions could be asked when necessary (Multimedia Appendix 5). Discussions were continued until no new themes and issues emerged. Discussions were conducted for approximately 45 to 60 minutes. All discussions throughout the sessions and consent for focus group discussion participation was recorded.

### Data Analysis

There were three rounds of usability testing done with redesigning of the app after each round.

The collected data were analyzed using Stata version 13. Findings are presented as descriptive statistics of frequencies. The null hypothesis for this study was there was no significant difference between mean SUS scores and mean time to complete a medication error report across the three sessions and between testers’ characteristics. Analyses of variance (ANOVA) and *t* tests were used to make a decision on whether to reject or accept the null hypothesis.

Verbatim reports of each recorded focus group discussion was transcribed by two independent research assistants. The verbatim reports were counterchecked for accuracy by the research assistants by switching their transcripts. The reports were then coded based on the seven theoretical themes by a researcher.

### Ethics Approval

This study was reviewed and approved by the National Medical Research and Ethics Committee of Ministry of Health, Malaysia (registration ID: NMRR-15-1445-27125[IIR]). The respondents were informed about the voluntary nature of participation. Participants were only served snacks during the focus group discussion and no other incentives were given. The results do not mention names of the participants. A formal letter of invitation to participate was issued to testers through their respective department heads.

### Funding

This research received no specific grants from any funding agency in the public, commercial, or not-for-profit sectors.

## Results

### Participant Characteristics

A total of 45 testers were available for testing, including 23 pharmacists, 13 doctors, and 9 nurses (Table 1). The ratio of selected pharmacists to doctors to nurses was 3:2:1. The majority of testers were female (35/45, 78%) and the mean age of testers was 36 (SD 9) years. Most testers were nonusers of the current medication error reporting system and nonexperts in medication errors.

### Quantitative Data

A total of 135 complete reports were successfully submitted by the testers (three reports per tester). Although all reports submitted were complete, there was deviation in answers provided in three domains: 12 of 135 (8.8%) in stage of medication process that error occurred, 8 of 135 (5.9%) in outcome of medication error, and 5 of 135 (3.7%) in drug involved in error (Table 2). Incorrect reports involving drugs were due to selection of the wrong drug form and only occurred in session 1. A note was included to inform users that drug names can be modified based on the drug involved in the error after session 1.

There was significant difference in mean SUS scores between the three sessions (*P*<.001; Table 2). The mean SUS score increased each session based on feedback from the testers. Experts rated lower SUS scores with a mean score of 72.8 (SD 2.4) compared to nonexperts (mean 80.1, SD 2.0, *P*=.03) and comparison of SUS scores between users and nonusers revealed no significant difference (Table 3).

**Table 1:** Characteristics of testers (N=45).

Characteristics		n (%)
**Gender**		
	Female	35 (78)
	Male	10 (22)
**Age (years)**		
	≤20 to <30	13 (29)
	≥30 to <40	17 (38)
	≥40 to <50	11 (24)
	≥50	4 (9)
**Profession**		
	Pharmacist	23 (51)
	Doctor	13 (29)
	Nurse	9 (0)
**Expertise in medication error**		
	Experts	18 (40)
	Nonexperts	27 (60)
**Users of current medication error reporting system**		
	Users	14 (31)
	Novice	31 (69)

**Table 2:** Quantitative data by sessions conducted.

Variables	Session 1	Session 2	Session 3	Overall	*P* value
Reports submitted, n (%)	45 (100)	45 (100)	45 (100)	135 (100)	—^a^
Complete reports, n (%)	45 (100)	45 (100)	45 (100)	135 (100)	—
Correct reports, n (%)	28 (62.2)	39 (86.7)	40 (88.9)	107 (79.2)	—
Drug name inaccurate, n (%)	5 (11.1)	—	—	5 (3.7)	—
Outcome of medication error incorrect, n (%)	4 (8.9)	2 (4.4)	2 (4.4)	8 (17.8)	—
Initial medication error process incorrect, n (%)	5 (11.1)	4 (8.9)	3 (6.7)	12 (26.7)	—
Time per report (mins), mean (SD)	6.5 (2.6)	7.1 (2.6)	6.5 (1.9)	6.7 (2.4)	.70
SUS score (%), mean (SD)	65.8 (10.2)	79.9 (4.5)	86.0 (3.8)	77.1 (10.8)	<.001

^a^Not applicable.

**Table 3:** Quantitative data of System Usability Scale (SUS) score by testers’ characteristics.

Variable		Mean (SD)	*P* value
**Age**			.10
	≤35 years	74.4 (12.3)	
	>35 years	79.7 (8.8)	
**Gender**			.50
	Female	77.8 (9.9)	
	Male	75.0 (13.8)	
**Expertise on medication error reports**			.03
	Expert	72.9 (10.2)	
	Nonexpert	80.1 (10.4)	
**Experience in current medication error reporting system**			.91
	User	76.9 (9.7)	
	Novice	77.3 (11.4)	

**Table 4:** Quantitative data on mean time per report submitted by testers’ characteristics.

Variable		Mean (SD)	*P* value
**Age**			.61
	≤35 years	6.90 (2.4)	
	>35 years	6.54 (2.3)	
**Gender**			.13
	Female	6.4 (2.5)	
	Male	7.7 (1.6)	
**Expertise on medication error reports**			.51
	Expert	7.0 (2.3)	
	Nonexpert	6.5 (2.4)	
**Experience in current medication error reporting system**			.02
	User	5.5 (2.0)	
	Novice	7.3 (2.3)	

Overall, the mean time to submit a report was 6.7 (SD 2.4) minutes. There was no difference in mean time to submit a report using the app between the three sessions (*P*=.70; Table 4). There was no difference in mean time for testers who were experts in medication errors to submit medication error reports compared to nonexperts (*P*=.51). However, users of the current medication error reporting system required a shorter mean time of 5.5 (SD 0.5) minutes compared to nonuser mean time of 7.3 (SD 0.4) minutes to submit a report (*P*=.02).

### Qualitative Analysis

Seven key themes were apparent from the group discussions: usability, visual design and layout, content, potential user engagement, security, validation of report, and other comments. The qualitative analysis will be summarized based on these themes.

#### Usability

In general, testers agreed that the app was easy to use and they required only a few tries to be familiarized with the functions in the app. However, several comments were provided by testers to improve navigation of MERA such as guided flow of upcoming field to fill, a “pop-up” box to proceed to the subsequent fill, a “next page” icon to proceed to the subsequent fill, and a summary of the filling guide at the beginning of the app. In the first session, nearly all the users struggled to identify how to add drug and medication process of error. It only required them to tap the bar, but it was not apparent to the testers.

The sequence of the questions needs to guided like numbering of the questions.

Doctor, male, nonexpert, novice

A summary in the beginning of the app explaining which part to “tap” or “click” to fill would be useful instead of having to trying on our own now.

Nurse, female, nonexpert, novice

After filling one data, the next data filling can appear in a “pop-up” manner so that users can know what to fill next.

Pharmacist, female, expert, user

I would prefer if it would be good if there is a next button to move to the next page. Now I am struggling to stroll up and down.

Pharmacist, female, expert, novice

When asked if they would require technical assistance to use the MERA, all unanimously agreed that would not be necessary.

Once you get the hang of it, it’s pretty easy to use.

Nurse, female, nonexpert, novice

Despite the challenges mentioned subsequently, all testers managed to submit complete reports in the first session of testing concluding that the app can be learned without guidance, but a guided app would ease users further.

#### Visual Design and Layout

The testers agreed that the design was simple and met its purpose. The majority of pharmacists understood the color selection was to match the color of the current medication error reporting form in the country. Although the font size was set at a standard 12 pixels, testers still preferred a larger font size. Testers also continued to comment on difficulty in identifying space to tap or click to fill in data and the design was improved based on their comments. When asked if the design flow of MERA was appropriate, most testers had positive comments and were satisfied with the design flow. Comments regarding the visual design and layout mentioned by testers are:

I would like the fonts to be bigger.

Pharmacist, male, expert, novice

If we want doctors to report, the font must be larger as most senior doctors are long and short sighted.

Pharmacist, female, expert, user

The colors are similar to the current medication error form from Ministry of Health: dark purple and light purple. I would prefer a contrast color in the rows that I need to fill in. I don’t know which place to key in data; this row should have an eye-catching color.

Pharmacist, female, expert, user

I would like to suggest that once data is keyed in, the row changes color indicating row already answered for the ease of users. This is because currently it is not obvious that you have filled that row.

Pharmacist, female, nonexpert, novice

**Figure 1:**
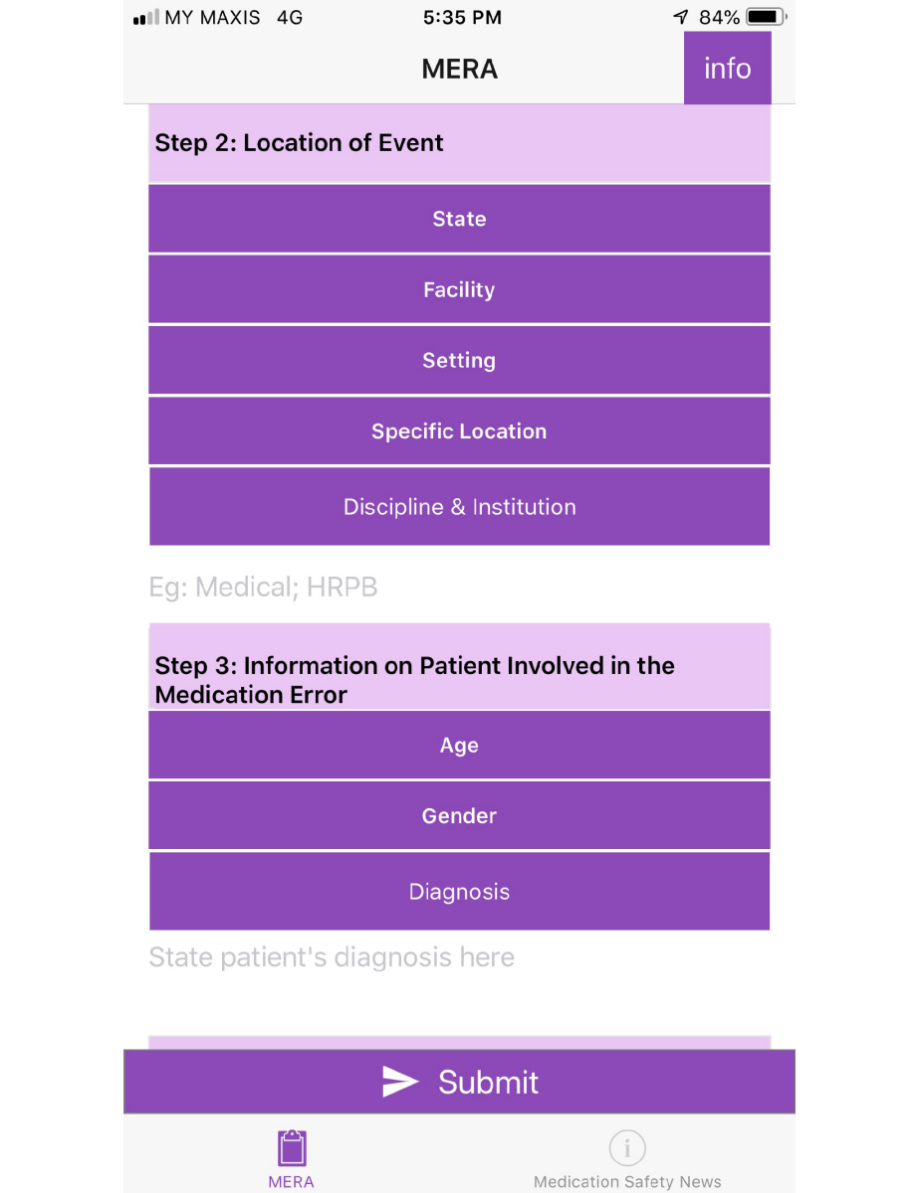
Screenshot of the final design of Medication Error Reporting Application (MERA) with each of the 12 steps of reporting medication error numbered.

**Figure 2:**
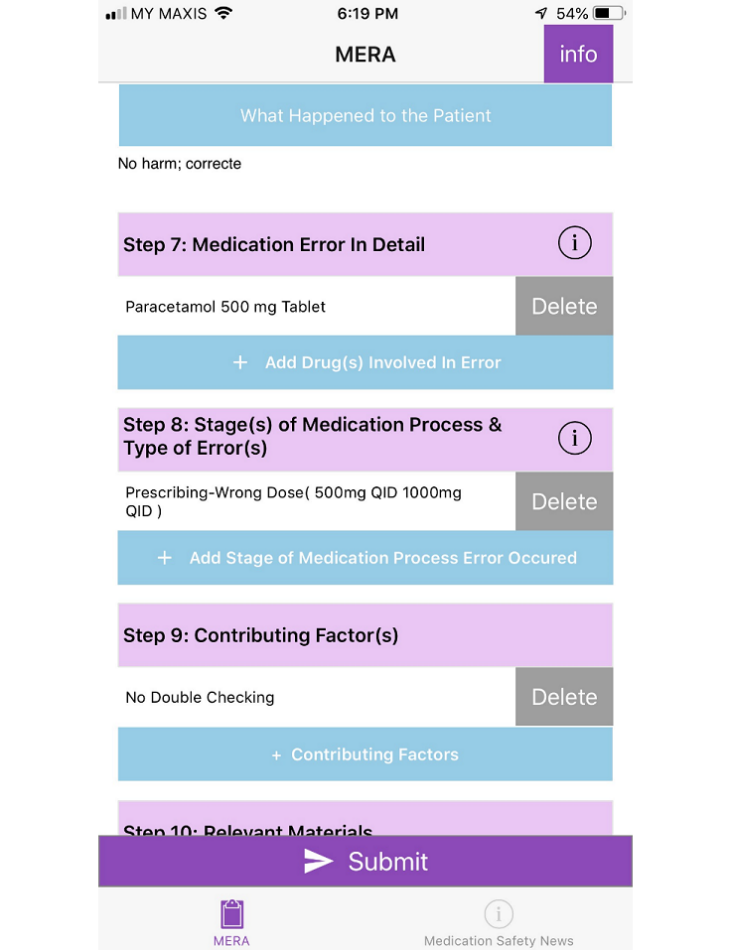
Screenshot of the final design of Medication Error Reporting Application (MERA). Purple bar changes to blue once tapped to fill report.

**Figure 3:**
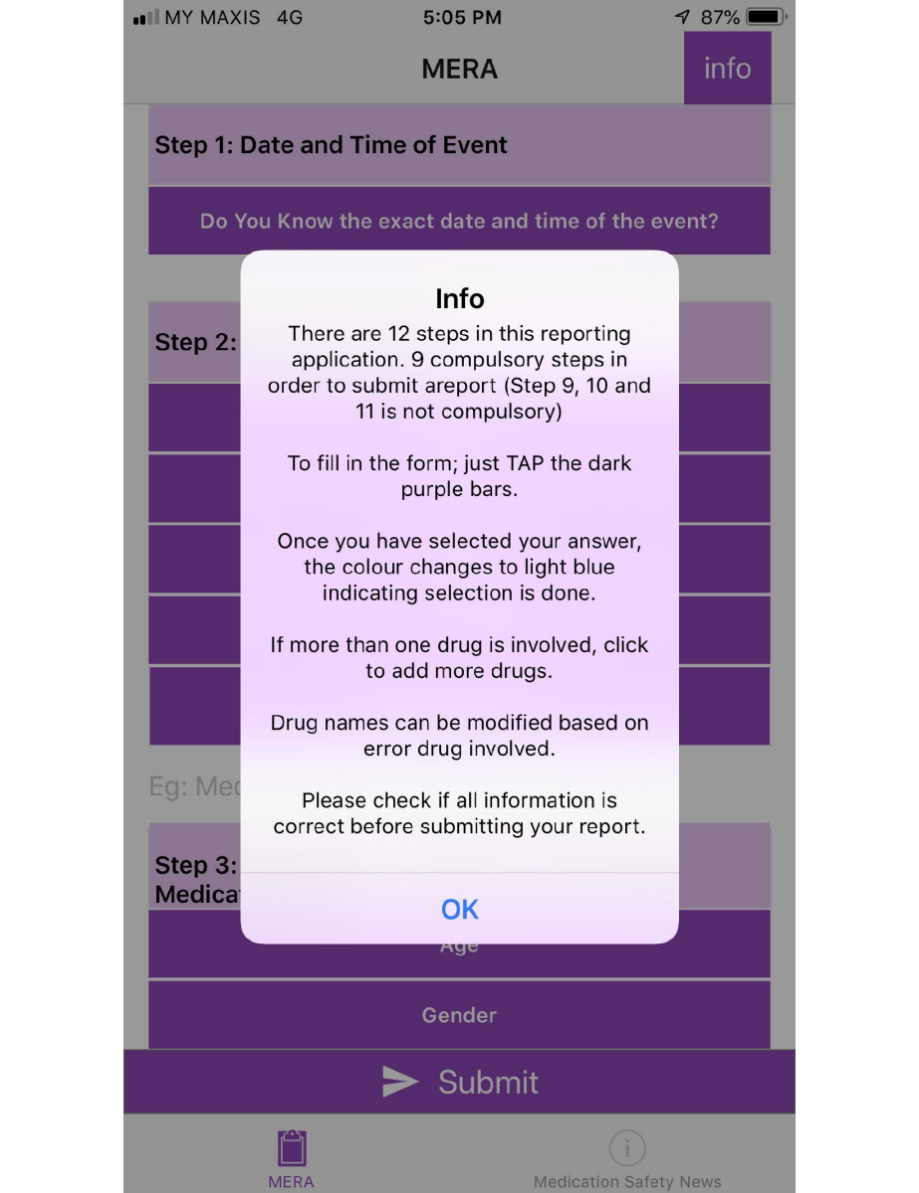
Screenshot of the final design of Medication Error Reporting Application (MERA) showing pop-up information of reporting instructions.

**Figure 4:**
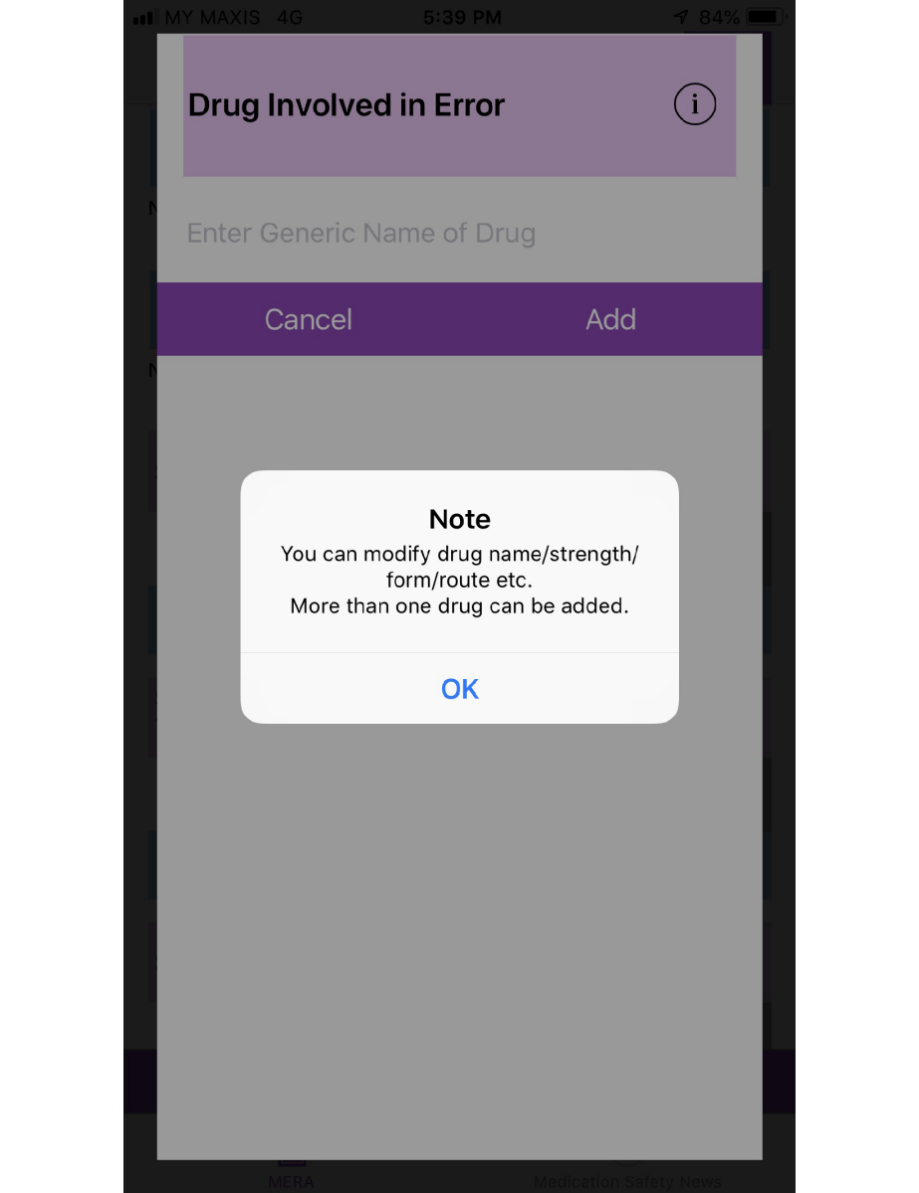
Screenshot of the final design of Medication Error Reporting Application (MERA) showing pop-up information about adding drug involved in error.

In order to address the comments and problems faced, app layout was modified to standardize the color scheme (dark purple) for rows that were required to be tapped to fill in data and each section was numbered as illustrated in Figure 1. Once the selection was done, the row changed to light blue as illustrated in Figure 2. Information about use of the app was located at the beginning of the app as illustrated in Figure 3 and information that drug names can be modified and more than one drug can be added was placed in the drug involved in error section as illustrated in Figure 4.

#### Content

There were many comments from the testers regarding content. Testers perceived that MERA itself was a simple, easy, and self-learnable app; however, filling in MERA required training especially on outcome of error, initial medication process that error occurred, and type of errors. Many testers requested to omit time of event.

##### Comments on Outcome of Error

Most of the testers, especially nonusers of the current medication error reporting system, were not sure how to code outcome of error. Pharmacists who were experts in medication error reporting commented that outcome of error in certain medication errors were difficult to determine and options were not provided to illustrate this in the medication error forms:

This is the first time I seen the outcome of error classification, maybe it’s my own ignorance. And I don’t know how to fill this column...Don’t get me wrong. It’s not the app; that’s straightforward. But the outcome of error is new at least to me.

Doctor, female, expert, novice

Can one of the options used in the outcome of error classification be UNKOWN as patients are not traceable after an error at times?

Pharmacist, female, expert, user

Understand why our doctor counterpart, have difficulties categorizing outcome of error because even pharmacists face similar difficulties especially if patient succumbs to death. It’s difficult to relate if medication error was the cause of death indirectly.

Pharmacist, female, expert, user

Errors that reach patient or did not reach patient is not included. It’s important to quickly identify near miss or actual error.

Pharmacist, male, expert, user

##### Comments on Type of Medication Error and Initial Medication Process That Error Occurred

The type of errors and initial medication process that error occurred appeared as jargon to doctors and nurses. Pharmacists in general understood the terms and some even requested more precise information. Here are some of the common cited issues as commented by testers:

The app can be used by everyone, so the process can’t be complex like type of error.

Doctor, male, expert, novice

App is easy but the data to key in especially type of error is not easy.

Doctor, male, nonexpert, novice

User-friendly terms maybe be useful.

Doctor, female, nonexpert, novice

Only the part to click the relevant medication error is not easy for me.

Nurse, female, nonexpert, novice

It depends how the tedious reporting person is; pharmacists are generally tedious and doctors are not when it comes to reporting...I am impressed that the app even has wrong formulation as an error category.

Pharmacist, female, expert, user

Transcribing process is missing as this is a common medication error process that is not captured in the current form and best included in MERA.

Pharmacist, female, expert, user

I think doctors would not be able to differentiate labeling, filling, or dispensing, so why not just stick to three major medication processes: prescribing, dispensing, and administration. We can identify the exact process during RCA [root cause analysis].

Pharmacist, female, expert, novice

I’m sure even my specialists are not familiar with the term used especially the terms such as labeling and preparation.

Doctor, female, nonexpert, novice

Some of the terms used are very pharmacy-based terms. That is why better to ask the pharmacist to report; they would better understand what to report.

Doctor, male, nonexpert, novice

A blank space to type briefly on medication error outcome and medication error event in the reporter’s own words was included. The administrator can then compare the filled-in outcome error and medication process with the brief description and correction can be made where appropriate. Time of medication error occurrence also had an option to select (weekday, weekend, public holiday, or on call) if users were not sure of exact time of medication error occurrence was also included.

#### Potential User Engagement

MERA is intended for all health care professionals and testers were asked if MERA could be engaged well by them; most doctors had negative responses compared to pharmacists.

If you need to engage doctors to use MERA, the app should be idiot-proof or else they might not use it.

Doctor, male, nonexpert, novice

If I am clinician and I have encountered medication error in my clinic, I might still not report using the app because I might forget the error.

Doctor, male, nonexpert, novice

I already have many apps on handphone already; I am not sure if I want another app.

Doctor, female, nonexpert, users

The MERA is smooth and fast and easier than manual for sure.

Pharmacist, male, nonexpert, novice

CME [continuous medical education] is definitely required before doctors and nurses can use MERA.

Pharmacist, female, expert, novice

Pharmacists were concerned about documentation of medication errors if reporting done via MERA because this would disrupt the statistics that is required for audit purposes:

In our government hospital setting, documentation for auditing is required. If we have manual and app; we of course go for the manual. This is for the purpose of the documentation part. That is why I don’t think we should use this app frequently. Unless we don’t need documentation, we can use apps only.

Pharmacist, female, expert, user

If it’s possible to print or save report; the app can be used.

Pharmacist, female, nonexpert, user

#### Security

At present, the app does not require any mode of registration before it can be used by users to ensure anonymity of users.

In each session, concerns about security of the app was questioned. Security of MERA was questioned in two aspects: security of medication error report data stored and news of the medication error reports. News on the app was suggested to be informative rather than just providing statistics on reported medication errors. A careful consideration on the feedback provided by MERA was recommended:

I assume...this app would be made available in AppStore and Google Store...it will be available to public as no registration is required. Public should not access to the statistics of reports in the News section of app.

Pharmacist, male, expert, user

The News section of app does it also post statistics of medication error reports? If so, the data can be misused if it falls in the wrong hands and can be misinterpreted. The MOH staffs can also misinterpret the statistics.

Pharmacist, female, expert, user

Any app can be hacked these days, even if its data secured to the MOH server.

Doctor, male, expert, novice

This app allows reporting error done by another staff; can this be misused?

Nurse, female, nonexpert, novice

#### Validation of Reports

The validation of reports posted some concern to testers. The current system is usually filled in manually and data are verified and the form is ensured complete by a local verifier. The medication error report then goes through a double verification process before the medication error report is accepted. A compulsory process for submission of medication error reports online requires identity of reporter:

How do administrators ensure that medication error reports via app is a genuine report?

Pharmacist, female, expert, user

What if more than one reporter reports the same medication error? How is this situation handled or identified?

Doctor, male, nonexpert, novice

If the reporter has selected the wrong selection by mistake; and report is submitted. This will be a problem, because as all required field is selected and filled, report is submitted.

Doctor, female, expert, novice

#### Other Features

Other features requested for MERA to make it more attractive were an indication of the compulsory questions to be filled and a pop-up to alert users if all data keyed in were accurate once Submit was tapped. This was to ensure that reporters were aware that once submitted, reports could not be amended. Users would like to save the reports in portable document format (PDF) to allow printing of reports to submit to any relevant authorities as required.

Another suggestion was to have some form of registration process to ensure that the app was only for health care professionals.

## Discussion

### Principal Findings

This usability testing using mixed methods provided vast informative input from testers in improving the MERA design. Improvements made to the design based on input provided clearly satisfied testers in the subsequent sessions as evidenced by the increasing SUS scores.

The app, from the discussion and tasks performed by the testers, proved to be simple and self-learnable. The design and layout were modified based on the useful insights from the testers.

The difficulty of reporting medication errors seems to lie in the three major parts of the medication error reporting as identified in this research: outcome of medication error, medication process when medication error initially occurred, and type of medication error. Outcome of medication error coding was an obstacle for all health care professionals, whereas medication process when medication error initially occurred and type of medication error were an obstacle to professionals other than pharmacists. Outcome of medication error used in MERA is the National Coordination Council for Medication Error Reporting and Prevention (NCC MERP) classification of outcome of error similar to the current reporting system in the country. A survey conducted among users of MEDMARX, an internet-based anonymous reporting system subscribed to by hospitals in the United States, reported kappa value of 0.61 (95% CI 0.41-0.81) among participants who rated error outcomes of 27 scenarios. This indicates only substantial interrater agreement among participants categorizing error outcome using NCC MERP. The overall percentage of participants that categorized error outcomes accurately based on the gold standard set was only 74% [16]. Testers in this study also faced the dilemma of correctly coding outcomes of medication errors resulting in incorrect reports submitted. It is essential therefore for MERA users to be trained regarding terms and classifications used in medication error reporting similar to the current reporting method. MERA app training may not require extensive training for reporting medication errors. The wide range of selection of initial medication processes when the error occurred and the type of error were maintained in the app. This wide range of categories are not available in the current manual reporting form. Incorporating a wide range of options for these two allows for quick verification of medication errors and report generation in the future. Incorrect submission of these two categories can be counterchecked with comments provided by reporters. Free text for outcome of error was included after the testing of the app. It also became apparent that doctors would rather have medication error reporting performed by pharmacists or nurses, as was reported in a recent literature review [7]. Doctors, nurses, and medical assistants who encounter medication errors in government hospitals and clinics in Malaysia must be prioritized for medication error reporting education. Nurses, and medical officers lack knowledge on medication error reporting process as mentioned in a qualitative study conducted recently [17].

In Malaysia, there are three reporting systems: adverse drug reaction reporting, medication error reporting, and incident reporting. All three reporting can be incorporated into one system. An app to report all three reporting can be considered for future use in Malaysia.

The major concern of reporting medication errors using a mobile phone app was validation of the reports. Should this mode of reporting be accepted in the future, similar verification methods as the current verification methods of medication errors can be employed, especially medication errors that have reached patients and caused harm. Each institution can have a local verifier who can trace patients based on location of error and basic patient details. A detail on location of error should be emphasized, such as a specific ward or clinic, and unique to the institution. Timely reports of actual medication error also should be emphasized during education sessions so that appropriate action can be undertaken, such as root cause analysis when required. Duplication of medication error reports also can be sorted out after cross-checking details of patients, drug involved in error, and type of error encountered by verifier.

Security of data stored was the major concern of the experts’ resistance to using the mobile app for reporting. News on the app that will be provided to users was another concern among experts as data obtained from new section can be subject to abuse by particular health personnel or institutions. This could be a major drawback in obtaining permission to use the app for reporting medication errors and this issue needs to be addressed appropriately. There were also similar concerns shown among testers in an app to report adverse drug reactions [18]

In regard to limitation of access to the app to health care personnel to safeguard information on medication reports to the public, various methods can be implemented which may incur cost and are not feasible at the moment for the purpose of this study. One such method is to assign a specific code that needs to be keyed in to launch the app. Codes will be issued to health care professionals by the administrative authorities of the institutions.

### Future Research

A mobile app to report medication errors has been successfully developed through usability testing and feedback of testers. Future work can be done to validate the use of MERA in a real clinical work setting to improve medication error reporting.

### Limitations

Features such as time of occurrence of medication error and details such as registration phase, consultation phase, admission phase, ward stay, or discharge phase can be incorporated into MERA. This would provide valuable information about which part of the health system is the weakest in the organization. This was not included throughout the design testing period and will be considered in the final design.

The study was not powered to analyze differences of SUS scores and time to report a medication error between experts and nonexperts, and users and novices.

Finally, the testers were all from one health institution; therefore, their views are not representative of personnel from other health institutions.

### Conclusions

MERA, the anonymous mobile app for reporting medication errors, can be used to report medication errors by various health care personnel conveniently with minimum user training. Security of the app, validation of reports, and abuse of feedback featured in the app seem to be of concern when using MERA. To encourage doctors and nurses in Malaysia to report medication errors, education on medication error reporting should be prioritized.

## Appendix

Multimedia Appendix 1 Type of medication error classification in Medication Error Reporting App (MERA).

Multimedia Appendix 2 Possible contributing factors appended in MERA based on an extensive literature search.

Multimedia Appendix 3 Task scenarios for usability testing.

Multimedia Appendix 4 System Usability Scale (SUS).

Multimedia Appendix 5 Question guide for focus group discussion.

## Abbreviations

MERA: Medication Error Reporting App
MOH: Ministry of Health
SUS: System Usability Scale
WHO: World Health Organization
