# Supplementary material for: Usability Testing of a Mobile App to Report Medication Errors Anonymously: Mixed-Methods Approach
Source: JMIR Hum Factors. 2018 Dec 21;5(4):e12232. doi: 10.2196/12232 (PMC6320434; doi:10.2196/12232)
Supplement: Multimedia Appendix 1 [file humanfactors_v5i4e12232_app1.pdf]

Prescribing Error: Commission Error
Wrong Drug / Drug Not Indicated / Wrong Dose / Wrong Strength / Wrong Formulation / Wrong Route / Wrong Frequency / Wrong Duration / Wrong Patient / Polypharmacy / Interaction Not Accounted / Contraindication
**Prescribing Error: Omission Error**
Drug Omitted / Dose Omitted / Strength Omitted / Formulation Omitted / Unit Omitted / Route Omitted / Frequency Omitted / Duration Omitted / Allergy Omitted / Weight Omitted / Age Omitted / Indication Omitted
**Administration Error: Commission Error**
Wrong Drug / Wrong Dose / Wrong Route / Wrong Formulation / Wrong Rate / Wrong Frequency / Wrong Duration / Wrong Patient
**Administration Error: Omission Error**
Drug Not Served / Dose Not Served
**Labelling Error**
Wrong amount to consume or administer / Wrong pre-post prandial advice / Incomplete instruction / Wrong instruction / No Instruction
**Filling Error**
Wrong Drug / Wrong Strength / Wrong Formulation / Wrong Amount
**Dispensing Error**
Wrong Drug / Wrong Dose / Wrong Strength / Wrong Formulation / Wrong Route / Wrong Frequency / Wrong Duration / Wrong Patient / Drug Missed Out
**Preparation Error**
**Monitoring Error**
**Compliance**
**Others**
