# Supplementary material for: Usability Testing of a Mobile App to Report Medication Errors Anonymously: Mixed-Methods Approach
Source: JMIR Hum Factors. 2018 Dec 21;5(4):e12232. doi: 10.2196/12232 (PMC6320434; doi:10.2196/12232)
Supplement: Multimedia Appendix 2 [file humanfactors_v5i4e12232_app2.pdf]

Prescription Related Factor(s)
o Illegible Handwriting
o Incorrect Transcribing
o Use of Non-Standard Abbreviation
o Use of Brand Name
o Adding Trailing Zero
o No Leading Zero
o Use U Instead of Units
o Use of µg Instead of mcg
o Verbal Order
Drug Related Factor(s)
o Sound Alike Drugs
o Look Alike Drugs
o Similar Packaging for Different Drugs
o Confusing Strength on Packaging
o Poor Readability of Printed Label on Drugs
o Inadequate Warning Labels on Drugs
o Labelling on Cap
Organizational System Factor(s)
o Tall Man Labelling Not Practiced
o No Double Checking
o High Concentration Drug Stored at Emergency Trolley
o No Pharmacist at Unit or Facility
o Lack of Information About Drug
o Verbal Order Allowed
Working Environment(s)
o High Workload
o Longer than 8 Hours of Work
o End of Shift
o Double Shift
o On Call
o Years of Repetitive Work
o Norm to Perform Task
o Seniors Unapproachable
Personnel Factors(s)
o Failure to Follow Standard Operating Procedures (SOP)
o Negligence
o Newly Transferred to the Facility / Unit
o First Year at Work
o First Posting at the Unit
o In Training
Patient Factors
o Patient not at bed
o Difficult Patient
o Patient has Hearing Impairment
o Mute Patient
o Language Barrier
Others. Please state
