# Supplementary material for: Usability Testing of a Mobile App to Report Medication Errors Anonymously: Mixed-Methods Approach
Source: JMIR Hum Factors. 2018 Dec 21;5(4):e12232. doi: 10.2196/12232 (PMC6320434; doi:10.2196/12232)
Supplement: Multimedia Appendix 3 [file humanfactors_v5i4e12232_app3.pdf]

Task #1

Mrs. A was on tablet levothyroxine 25 mcg OD in the ward. She was newly diagnosed as having hypothyroidism. She had been planned for discharge and collected her medication from the outpatient pharmacy. During her next follow up on 20th March 2017 at the Medical Specialist Clinic of Hospital B, which was 2 weeks later after discharge, she was noted having tachycardia and her heart rate was 150 bpm. Upon history taking by specialist, it was noted by him that patient's medication envelope was labeled as "Take 1 tablet, once a day" and filled with tablet levothyroxine 100 mcg. The pharmacy staffs involved (pharmacy assistant who filled and pharmacist who dispensed) were informed of the medication error by the specialist. Patient did not have other complaints and was sent home.

Task #2

On January 7th 2017, specialist ordered syrup trimethoprim 5mg OD for a 2.5 kg baby, day 1 of life in a pediatric ward (Hospital C). House officer (HO) transcribed wrongly in BHT and medication chart as 50mg OD (20mg/kg/day). **Prescribed 10 times higher than normal dose.** The prescription reached 24 hours Pharmacy. Pharmacist did not screen the prescription but passed the prescription to Provisional Registered Pharmacy (PRP) (assuming prescription will be screened by PRP). PRP did not check the appropriateness of dose and went about to prepare syrup based on prescription extemporaneously. Worksheet prepared by PRP was countered checked by the same pharmacist. Syrup trimethoprim 50mg/6ml was prepared and sent to ward at around 9.00 pm.
In the ward, nurse administered the medication as prescribed 6ml (50mg) daily as prescribed without noticing the large amount requiring for a neonate at 9.10pm.
Prescription error was detected during routine ward rounds the following morning by ward pharmacist. Baby was discharged on 9th January 2016 without any adverse events.

Task #3

Mr. D was admitted to the surgical ward at night (3rd June 2017) due to motor vehicle accident (MVA) and was prescribed IV tramadol 50mg stat and three times per day at 11pm. During ward round in the morning (4th June 2017), Mr. D complained of severe pain. Ward pharmacist following rounds checked his medication chart and noticed IV tramadol was not administered (administration column not signed). The involved nurse admitted that she did not to serve the medication due to improper pass over from last night shift.
