# Supplementary material for: Usability Testing of a Mobile App to Report Medication Errors Anonymously: Mixed-Methods Approach
Source: JMIR Hum Factors. 2018 Dec 21;5(4):e12232. doi: 10.2196/12232 (PMC6320434; doi:10.2196/12232)
Supplement: Multimedia Appendix 4 [file humanfactors_v5i4e12232_app4.pdf]

System Usability Scale

	Strongly Disagree 1	2	3	4	Strongly Agree 5
1. I think that I would like to use this system frequently					
2. I found the system unnecessarily complex.					
3. I thought the system was easy to use.					
4. I think that I would need support of a technical person to be able to use this system.					
5. I found the various functions in this system was well integrated.					
6. I thought there was too much inconsistency in this system					
7. I would imagine that most people would learn to use this system very quickly					
8. I found the system very cumbersome to use					
9. I felt very confident using the system.					
10. I needed to learn a lot of things before I could get going with this system					
