# Supplementary material for: Usability Testing of a Mobile App to Report Medication Errors Anonymously: Mixed-Methods Approach
Source: JMIR Hum Factors. 2018 Dec 21;5(4):e12232. doi: 10.2196/12232 (PMC6320434; doi:10.2196/12232)
Supplement: Multimedia Appendix 5 [file humanfactors_v5i4e12232_app5.pdf]

- How would you describe the medication error reporting app (MERA) that you had opportunity using just now?
- Which part of the MERA that you found difficult based on your experience submitting reports?
- What particular positive or negative thing(s) that caught your attention about the MERA?
- What are your comments on the design and layout of the MERA?
- Regarding the flow of the content of MERA; is there any particular issues you would like to share?
- What you think of learnability of the MERA?
  Prompts: Do you think you need Intelligence Technology (IT) assistance to use MERA?
  Prompts: Do you think your colleagues would need Intelligence Technology (IT) assistance to use MERA?
- Is there anything that you would suggest to make the improve the app?
  Prompts: Is there any feature missing in MERA to report medication errors?
  Prompts: Is there any unnecessary feature in MERA to report medication errors?
- What is your opinion about the Medication Safety News in MERA?
- Do you think you or your colleagues would use MERA to report medication errors in the future?
  Prompts: Why wouldn't you or your colleagues use MERA?
  Prompts: Why would you or your colleagues use MERA?
- Are there any other comment(s) regarding MERA that you would like to share that we have not covered here?
